# Why network approach can promote a new way of thinking in biology

**DOI:** 10.3389/fgene.2014.00083

**Published:** 2014-04-08

**Authors:** Alessandro Giuliani, Simonetta Filippi, Marta Bertolaso

**Affiliations:** ^1^Department of Environment and Primary Prevention, Istituto Superiore di SanitàRome, Italy; ^2^Nonlinear Physics and Mathematical Modeling Laboratory, University Campus Bio-Medico Rome, Italy; ^3^International Center for Relativistic Astrophysics, University Campus Bio-MedicoRome, Italy; ^4^Institute of Philosophy of Scientific and Technological Practice, Faculty of Engineering, University Campus Bio-MedicoRome, Italy

**Keywords:** complex networks analysis, middle-out approaches, mesoscopic descriptors, systems biology, graph theory

## Abstract

This work deals with the particular nature of network-based approach in biology. We will comment about the shift from the consideration of the molecular layer as the definitive place where causative process start to the elucidation of the among elements (at any level of biological organization they are located) interaction network as the main goal of scientific explanation. This shift comes from the intrinsic nature of networks where the properties of a specific node are determined by its position in the entire network (top-down explanation) while the global network characteristics emerge from the nodes wiring pattern (bottom-up explanation). This promotes a “middle-out” paradigm formally identical to the time honored chemical thought holding big promises in the study of biological regulation.

## INTRODUCTION

The classical form in which biological systems are described (being they metabolic charts, gene expression regulation pathways, protein–protein interaction maps, intercellular connections, food webs, and so forth) corresponds to a set of nodes linked by edges in which the nodes are the basic elements of the described system (genes, proteins, metabolites, cells, and so forth) and the edges connecting them some rules of the kind “is transformed into” or “is increased by” or, more in general “is correlated with.”

The figures normally present in books and scientific papers implicitly consider these pathways as linear causative chains in which a signal starting from a molecular perturbation, after a sequence of “if-then” events, emerges as a biological end-point (Tun et al., 2011). Normally these processes are referred as “cascades” provoking a progressive amplification of the initial stimulus (MacFarlane, 1964).

This style of reasoning considers biological systems having a strictly hierarchical architecture going from molecular to whole organism level and in which the ultimate causative layer is the most microscopic one, i.e., the molecular level (genes).

The widespread recognition of the limitations of this purely bottom-up way of reasoning [e.g., the problems encountered in genome-wide-association-studies (GWAS), see McCarthy et al., 2008] is in general ascribed to lack of sufficient statistical power of the study and to the need of more sophisticated analyses. The recognition of alternative “ultimate” explanation levels is in general referred to as “epigenetics” (Jiang et al., 2004).

The development of high throughput “omics” methodologies in which thousands of variables (genes, proteins, metabolites) are measured in parallel on the same statistical units (biological samples) made the graphs corresponding to the “perceived” regulation networks sketched in the usual “box-and-arrow” style to become larger and larger and urgently asked for some kind of global analysis in order to get rid of their wild multiplicity.

Considering the graph as a system of differential equations in which an entering stimulus, correspondent to a modification of a peripheral node of the network, is progressively processed according to the wiring architecture and kinetics constraints is the most powerful representation. In the case of biological systems this avenue of research is severely hampered by a lot of problems like the difficulties in parameter estimation (Gutenkunst et al., 2007) overfitting (Sun et al., 2012), lack of stationarity (Donner et al., 2011).

For these reasons many authors preferred a purely topological approach to the analysis of biological networks (Nordling et al., 2007; Dehmer et al., 2013) considering the presence of a link between two nodes as a pure yes/no binary relation and limiting themselves to statistical descriptions making use of the so called graph invariants (Watts and Strogatz, 1998). Graph invariants are statistical descriptors of networks relying on the simple count of nodes and edges with no reference at the nature of each node, enabling the analyst to identify crucial elements of the network or to highlight specific features of the entire network architecture responsible for some aspects of the studied system behavior (Watts and Strogatz, 1998).

In a recent work Dehmer et al. (2013) demonstrated the efficiency of graph invariants derived by the adjacency matrix coming from the gene expression signatures of different patient samples in predicting the different disease states with no explicit reference to the specific nature of the involved nodes (genes).

The existence of an autonomous level of analysis, independent of the specific properties of the elements and directly deriving from the wiring pattern is at the basis of the so called Tellegen theorem (Tellegen, 1952; Mikulecki, 2001) stating the thermodynamics of each system has two complementary but distinct contributions: the constitutive laws for the network elements and the network topology. The use of constitutive laws for the networks elements is the usual way we tackle natural phenomena, Tellegen demonstrated (Tellegen, 1952) the topology or connected pattern of these elements constitutes an independent reality about the system (Mikulecki, 2001). The same topology can be realized for an infinite variety of network elements giving rise to shared universal properties.

The importance of purely topological properties is widely accepted in protein science, where protein contact networks allow to derive crucial functional features of the studied systems (Di Paola et al., 2013) but it is still in its infancy in the field of biological regulation.

We will show in the next how this way of reasoning can by no means be considered as a new approach being at the basis of chemical thought since the widespread use of structural formulas (a particular form of graph) in organic chemistry around 80 years ago allowing to define a generalized “Graph-Energy” (Gutman and Zhou, 2006) analogous to the Huckel Molecular Orbital Theory used by chemists to approximate Π-electron energies (Gunthard and Primas, 1956).

### GRAPHS: A “CHEMICAL” CONCEPT

Network graph-theoretical approaches are located half-way between bottom-up and top-down approaches focusing on the relation between the elements of the studied phenomenon. We can roughly describe the network approach as the answer to the question “What can we derive from the sole knowledge of the wiring diagram of a system?”

**FIGURE 1:**
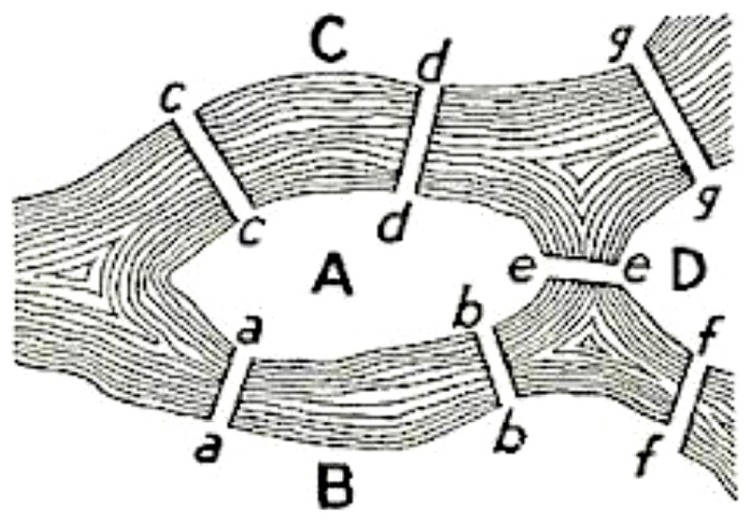
**The Konigsberg bridge problem: the seven bridges (edges) extremities are indicated by letters (nodes)**.

The classic Konigsberg bridge problem introduced graph theory in 18th century. The problem had the following formulation: does there exist a walk crossing each of the seven bridges of Konigsberg exactly once? The solution to this problem appeared in “Solutio Problematis ad geometriam situs pertinentis” by Eulero (1741). This structure was called a graph and this was the first time a problem was codified in terms of nodes and edges (**Figure 1**).

The problem was to find a walk through the city that would cross each bridge once and only once. The islands could not be reached by any route other than the bridges, and every bridge must have been crossed completely every time; one could not walk halfway onto the bridge and then turn around and later cross the other half from the other side. The walk need not start and end at the same spot. Eulero proved that the problem has no solution. The interest of the Eulero demonstration lies in the fact he considered as the only important feature for the solution the sequence of bridges crossed. He formalized the problem in terms of nodes (land masses) and edges (bridges) connecting the nodes. The resulting mathematical structure is called a graph.

**FIGURE 2:**
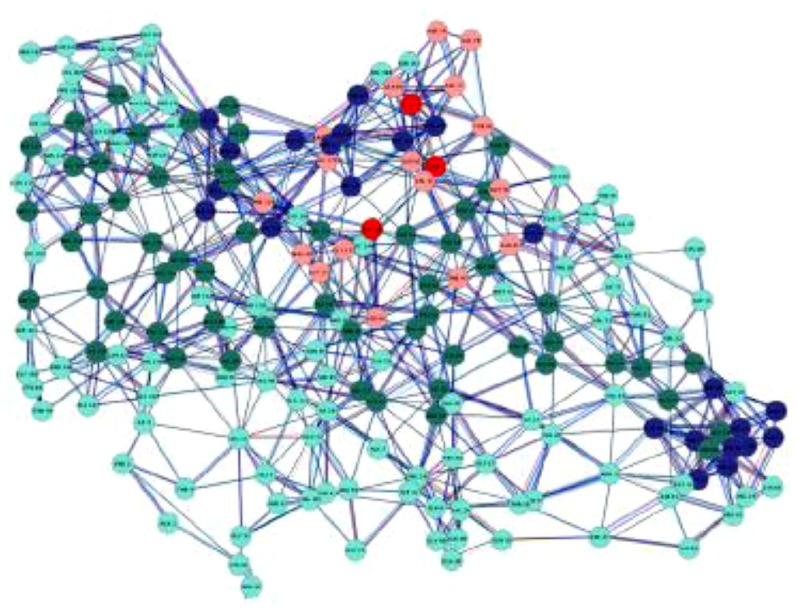
**A protein contact network (PCN): this is a complex graph in which each node corresponds to an aminoacid residue and each edge to a physical contact between two residues**. The nodes are variously colored according to aminoacid chemico-physical features (Di Paola et al., 2013). The chemico-physical features can be made to correspond to the Tellegen’s constitutive laws, while the wiring pattern has an autonomous status.

More in general, a graph G is a mathematical object used to model complex structures and it is made of a finite set of vertices (or nodes) V and a collection of edges E connecting two vertices (**Figure 2**).

Graphs can be defined by descriptors located at local (single nodes), global (entire network), and mesoscale (clusters of nodes, optimal paths) levels. Thus we can compute the degree of each node (how many links are attached to a given node) that is a local, microscopic characteristic or we can compute the so called “average shortest path” or “characteristic length” of a graph corresponding to the average length of minimal paths connecting all the node pairs (this corresponds to a mesoscopic feature of the system) or the general connectivity of the network (A global feature; Watts and Strogatz, 1998; Di Paola et al., 2013).

It is important to stress these different views are strictly intermingled among them, given they derive from the same basic representation (the graph) so that any view influences (and in turn is influenced) by all the others. The necessary (and natural) interaction of different level views is called “middle-out” approach to stress the fact the interest is focused on the mesoscopic level, i.e., on the pattern of between elements relation and not on the fundamental features of the constituting elements (Csermely et al., 2005).

The science that was mostly influenced by this “naturally systemic” view is chemistry that uses since decades the most widespread (and effective) graph formalization: the structural formula (Di Paola et al., 2013). An hydrogen atom embedded into methane molecule has different features than the same hydrogen atom of a water molecule: e.g., the hydrogen in the water molecule has a partial positive charge much greater than the methane hydrogen for the greater electronegative character of oxygen with respect to carbon atom. This is a clear example of top-down causation: the properties of the most basic level (atom) depends on the features of the entire system (molecule). At the same time both methane and water molecules derive their features from the constituent atoms (bottom-up causation). Stressing the two “directions of causality” is in any case out-of-scope, because the chemical graph incorporates both into a global systemic reasoning made it possible by the structural formula or chemical graph.

If we shift to more complex formulas of organic molecules we can appreciate the richness of the possibilities offered by this approach by the way thousands of different quantitative features of the molecules can be directly derived from structural formulas so that, strictly speaking, properties like solubility, melting point, molar refractivity, partition coefficients can be considered as graph descriptors (Todeschini and Consonni, 2008).

Organic chemistry is thus a perfect example of the power of middle-out approach: a chemical graph (structural formula) allows for deriving functional properties of the studied object (chemico-physical and reactivity features), in other words, a “semantics” emerges from a purely syntactical approach (molecular graph). In the case of chemistry, the existence of very powerful “network-based-theories” is a crucial ingredient of the success of network approaches. As a matter of fact the so called “energy of a graph” E(G) can be defined on a purely topological basis as the sum of the absolute values of the eigenvalues of its adjacency matrix (de Abreu et al., 2010), theoretical chemists did know since decades (Gunthard and Primas, 1956) that, under some reasonable assumptions the graph energy was coincident with the total Π-electrons energy according to Huckel molecular orbital theory.

The marked superiority of the simple consideration of molecular graphs over more sophisticated methods in the prediction of biological activity of drugs is an important proof of the power of the graph-theoretical approach in medicinal chemistry (Bender and Glen, 2005).

Is it possible, at least in perspective, to apply this style of reasoning when going into the more “fuzzy” biological world? The work of Denis Noble about the absence of a single privileged level of causation (Noble, 2012) and the need to go from a molecular to a modular approach set forth by Hartwell et al. (1999) go along this direction. In the following we will sketch some operative examples of this kind of approach, focusing on the use of graph-theoretical methods.

### GOING INTO BIOLOGY

A very simple biological proof of the efficiency of the “network-style” of reasoning, beside the much more sophisticated and deep Dehmer et al. (2013) analysis quoted in the introduction, is the prediction of lethal mutants in yeasts by the graph analysis of their metabolic network (Palumbo et al., 2005, 2007).

As we stated before, all the properties relative to each node (edge) must be derived only by its pattern of relations and thus by its peculiar location in the complete graph. In (Palumbo et al., 2005, 2007) the authors checked for the possibility to derive, from purely topological information on the metabolic network of yeast (*Saccharomyces cerevisiae*), the lethal character of genetic mutations. A metabolic network can be considered as a graph having enzymatic reactions as edges and metabolites as nodes. Since an enzymatic reaction is catalyzed by one or more enzymes, an edge can also represent the enzymes involved in the reaction. The experimental knock-out of an enzyme corresponds to the elimination from the network of the edge (or edges since the same enzyme can catalyze different reactions) corresponding to that particular enzyme (Palumbo et al., 2005). If it is possible to pick up a connectivity descriptor able to unequivocally define essential enzymes (those enzymes whose lack provoke the yeast death) we can safely assume the biological relevance of the metabolism “wiring structure”, irrespective of the specific nature of the involved enzymes.

In the case of yeast metabolic network, the analysis of 36 lethal mutations out of the 412 relative to enzymes involved in metabolism, reported in the Stanford repository (http://www-sequence.stanford.edu/group/yeast_deletion_project/deletions3.html) and in Jeong et al. (2003) and cured by Ma et al. (2004), allowed the authors to discover that the enzymes corresponding to lethal mutations, when deleted, prevent the connections between the separate nodes (Palumbo et al., 2005, 2007). No alternative path is available to connect the separate nodes and this mesoscopic feature based on paths along the network explains the essential character of each specific mutation on a pure topological basis.

This “essentiality-by-location” mesoscopic principle equating the lethal character of a mutation to the lack of an alternative path in the network, was confirmed in (Palumbo et al., 2007) demonstrating that a double mutation involving two enzymes that *per se* are not essential acquires essentiality and then causes the death of the organism, if the double knock-out provokes the “lack of alternative path” condition. The arising of lethality by the summation of two non-lethal events derives from the existence of a global metabolism architecture and thus cannot be inferred by going in depth into the nature of the two enzymes, in other words is a collective emergent property of the network system (Mikulecki, 2001; Giuliani, 2010).

The lack of exceptions to the “lack of alternative path rule” seems to rule out the existence of lethal mutations deriving by poor kinetics, but this is might be a too strong deduction. Indeed it is worth noting that the analyzed Stanford repository refers to experiments carried out in the same experimental conditions and thus eliminating all the “real life” well known difficulties that make the phenotypic effects of a given mutation strongly context dependent so that even relatively minor variations of nutrients, pH, temperature can exert dramatic effects.

The reported study must be intended as a proof-of-concept of the possibility to observe the “pure topology” properties devised by Tellegen theorem in a biological context, in any case by no means can we consider strictly topological approach as the obliged way for middle-out approaches in biology.

The contemporary presence of “hard wired” topology driven (presence/absence of a link irrespective of its strength is the main driver) and “transient functional” (kinetics plays a relevant role) relations in biological regulation is at the basis of another very interesting application of the “Middle-Out” (starting from the relations) approach.

In their very interesting work, Han et al. (2004) modeled the correlation dynamics of the mutual relation between hubs (proteins engaging a very high number of relations with other proteins, i.e., network elements with a very high node degree) and their partners by using messenger RNA expression profiles.

The authors examined the extent to which hubs in the yeast interactome are co-expressed with their interaction partners: for each hub they computed the average Pearson correlation coefficient (APCC) between the hub mRNA expression and its neighbors and found that APCC followed a bimodal distribution clearly evidencing two distinct hub populations. They called “party hubs” those nodes that were highly correlated as for expression with their partners (high values of APCC). By contrast, they called “date hubs” these nodes characterized by lower APCC values. Looking at particular protein-protein interactions of “party” and “date” hubs the authors discovered high APCC correspond to permanent interactions while “date” hubs correspond to transient interactions (see also Blasi et al., 2005). Moreover, the authors (Han et al., 2004) demonstrated a link between this hub classification and the network tolerance against node breakdown. Scale-free networks are particularly resistant to random node removal (failure) but are extremely sensitive to targeted removal of hubs (attack; Jeong et al., 2001). Han et al. (2004) showed that the removal of party hubs did not affect the network characteristic path length (and consequently the efficiency of network integration), as it happens in the case of failures: conversely when deleting date hubs, the effects were similar to those expected in the case of targeted attacks with a dramatic increase in characteristic length and thus a neat decrease in network communication efficiency.

## CONCLUSION

The specific role of Systems Biology is, in our opinion, to integrate mainly mechanistic biological thinking with a relational paradigm analog to chemical thought. This positive influence can only be obtained by means of the conscious use of network-based approaches, given the graphs have “embedded in their intimate nature” the co-existence and mutual interactions of different explanation layers. This interaction stems from the computation of graph invariants and thus is independent of any specific theory or assumption on the studied phenomenon.

While there are many examples of complex network approaches in the description of biological systems, what in our opinion is lacking and could constitute a new frontier is the conscious development of a network-based statistical mechanics approach (the Dehmer et al., 2013 paper is a starting point) considering autonomous “network biomarkers” irrespective of the “constitutive laws” of the constituting elements: we are convinced this kind of approach could make us to appreciate completely new and unexpected “biological organizational laws”.

## Conflict of Interest Statement

The authors declare that the research was conducted in the absence of any commercial or financial relationships that could be construed as a potential conflict of interest.
